# Regional estimation by oxygen isotope ratio analysis using tooth enamel of Japanese individuals

**DOI:** 10.1093/fsr/owaf024

**Published:** 2025-09-05

**Authors:** Kaisei Ono, Hidetoshi Someda, Masatsugu Hashimoto, Yasutaka Nakamura, Ichiro Tayasu, Chikage Yoshimizu, Noboru Ishikawa

**Affiliations:** Department of Forensic Odontology and Anthropology, Tokyo Dental College, Tokyo, Japan; Department of Social Affairs and Relief Bureau, Ministry of Health, Labour and Welfare, Tokyo, Japan; Department of Forensic Odontology and Anthropology, Tokyo Dental College, Tokyo, Japan; Department of Forensic Odontology and Anthropology, Tokyo Dental College, Tokyo, Japan; Research Institute for Humanity and Nature, Kyoto, Japan; Research Institute for Humanity and Nature, Kyoto, Japan; Department of Forensic Odontology and Anthropology, Tokyo Dental College, Tokyo, Japan

**Keywords:** forensic sciences, forensic odontology, tooth, enamel, regional estimation, isotope analysis, stable isotope, oxygen isotope, carbon isotope

## Abstract

In the field of forensic sciences, human teeth are used to identify individuals in cases involving unidentified bodies. In recent years, isotope analysis of tooth enamel has been increasingly employed to estimate birth year and place of birth. The enamel is formed between the prenatal period and childhood, and after the tooth crown is complete, it does not undergo additional growth. Therefore, the oxygen isotope composition of the enamel bioapatite is significantly influenced by the environmental conditions during these periods, including dietary habits and tap water consumption. In this study, we aimed to predict the places of birth of 65 Japanese individuals, whose places of birth were known, by analysing the oxygen isotope ratios in carbonates in the enamel bioapatite. The oxygen isotope ratio in bioapatite varied from a maximum value of −3.4‰ to a minimum of −8.76‰, indicating lower and higher values in cold and warm areas, respectively. Furthermore, a correlation was observed between the oxygen isotope ratios and the latitudes and average annual temperatures of the place of residence during enamel formation (correlation coefficients: −0.84 and 0.81, respectively). Oxygen isotope analysis of enamel bioapatite can help in determining the environmental conditions in the place of residence during enamel formation. Overall, oxygen isotope analysis can be useful in predicting the place of residence during enamel formation of individuals in Japan.

**Key Points**
 

- Oxygen isotope ratios in tooth enamel bioapatite were analysed to determine the birthplaces of Japanese individuals.
- The oxygen isotope ratio correlated with the latitude and average annual temperature of the place of residence during enamel formation.
- Oxygen isotope analysis can help in estimating the place of birth of individuals in Japan.

## Introduction

The tooth is preserved for a long time after death and is less susceptible to decay and contamination than bones. Additionally, hard tissue components remain stable over a long term [1, 2]. The morphological characteristics of teeth and dental arches, as well as DNA analysis results on cementum and dental pulp, are used to estimate demographic factors such as population affinity and birthplace [3, 4]. Furthermore, in recent years, based on the bomb pulse, artificial radiocarbon in teeth has been used to estimate the birth year and place of birth [5–9].

Isotopes are elements with the same atomic number but different numbers of neutrons in their atomic nuclei. The isotope ratio, which represents the proportions in which the isotopes exist, has been reported to vary slightly due to environmental differences, even within the same organism or substance [10]. Isotopes can be categorized into two types: radioisotopes, which are radioactive and prone to decay, and stable isotopes, which are not radioactive and do not undergo decay. In particular, in the field of environmental sciences, the ratio of stable oxygen isotopes (referred to as the “^18^O/^16^O ratio”) is used for climatic pattern estimation. In archaeology, carbon and nitrogen are utilized to analyse animal diets because they are influenced by the food animals consume [11–13]. Oxygen isotope ratios and strontium isotope ratios, on the other hand, are used for the estimation of mobility as they are attributed to the dynamics of bedrock and water [14–16].

*Via* a continuous cycle, water from the sea evaporates to form water vapour, turns into clouds, precipitates as rain, flows as groundwater and stream water, and returns to the sea (water cycle). Water containing lighter isotopes (^16^O) tends to evaporate more easily than that containing heavier isotopes (e.g., ^18^O). When water vapour evaporates from the sea, the ratio of ^16^O to all oxygen atoms in the water vapour becomes higher than the ratio in sea water. However, if the rate of evaporation increases, the proportion of the heavier isotope ^18^O, which evaporates more slowly, also increases. Therefore, in areas with faster evaporation rates, such as low latitudes and warm areas, rainfall with a higher ratio of ^18^O relative to ^16^O is expected compared to that in high latitudes and cold areas with slower evaporation rates (latitude effect; temperature effect). Furthermore, in areas with higher elevations, clouds with an increased proportion of evaporated water from inland water sources and rivers are formed. This results in rainfall with a low ratio of ^18^O relative to ^16^O (altitude effect). Due to these characteristics, area maps (i.e., isoscapes) have been designed that illustrate the variation in oxygen isotope ratios [10, 17, 18].

The enamel is formed between the prenatal and childhood periods, and after tooth crown completion, it does not undergo additional growth. Therefore, the *δ*^18^O present in apatite is significantly influenced by precipitation, groundwater, and other components of the water cycle during these periods, as the oxygen isotope present in these components is incorporated into the body *via* tap water [15, 19]. Examination of the oxygen isotope ratio in carbonate in the enamel apatite would help in the estimation of the geographical environment at the time of enamel formation based on these characteristics [15, 19, 20].

In this study, we aimed to estimate the place of residence during enamel formation within Japan by examining the oxygen isotope in carbonates in the enamel bioapatite. Furthermore, since a shift in the isotope ratios of ^18^O was observed upon reanalysis of tooth enamel following 10 years of storage [21], it is important to determine whether such changes arise even during a short time period, which is more relevant to practical cases. Therefore, we investigated the change in isotope ratios following 1 month of storage in this study.

## Materials and methods

### Samples

The samples comprised one tooth each from 65 individuals born in Japan; these teeth (third molars, which have a tooth germ formation period of 11–12 years after birth) [9, 22] were stored at the National Defense Medical College Research Institute. The subjects consisted of 58 males and seven females, ranging in age from 20 to 52 years, with a mean age of 31 years. The study protocol complied with the codes of ethical practice of the Tokyo Dental College and National Defense Medical College. All the study procedures and the use of human samples were approved by the medical and ethical committees of the Tokyo Dental College (approval number: 1127) and National Defense Medical College (approval numbers: 2846-1 and 2846-2). This study followed the ethical standards outlined in the Declaration of Helsinki, a set of guidelines established by the World Medical Association. Informed consent was obtained through written procedures in accordance with these ethical principles. Furthermore, the tooth enamel formation period for each sample ranged from 1960 to 2000. However, participants who may have moved across residential areas at least once were excluded. The geographical distribution of the samples is shown in Table 1.

**Table 1:** Geographical distribution and number of tooth samples by region in Japan (*N* = 65).

**Area**	**Prefecture**	**Number of samples**
Hokkaido	Hokkaido	5
Tohoku	Aomori Iwate Miyagi	1 1 3
Kanto	Ibaraki Saitama Tokyo Kanagawa	2 4 1 2
Hokuriku	Toyama Ishikawa Niigata	1 1 3
Tokai	Gifu Aichi Shizuoka Mie	1 3 2 1
Kinki	Nara Osaka	1 4
Chugoku	Hiroshima Tottori Shimane	2 2 1
Shikoku	Tokushima Kagawa Ehime	2 2 1
Kyushu	Kumamoto Fukuoka Saga Nagasaki	2 4 1 1
Okinawa	Okinawa	11

### Sample preparation

To remove the surface stains, the enamel surface was ground using a dental stainless-steel round bur (Shofu, Japan). Subsequently, the enamel over the entire circumference, excluding the occlusal surface, was ground, and the enamel powder (12–15 mg) was collected. The collected enamel powder was treated with 1 mL of 3% H_2_O_2_ (Tama Chemicals Co., Ltd, Kawasaki, Japan) (JIS special grade) to remove organic contaminants. After 24 h, the mixture was centrifuged using a tabletop centrifuge (Kubota Corporation, Osaka, Japan), and the supernatant was removed. Next, 1 mL of double distilled water (DDH_2_O) (Merck, Tokyo, Japan) was added, and the mixture was centrifuged. This process, including the removal of the supernatant, was repeated twice. Subsequently, to remove secondary carbonates, 1 mL of 0.1 mol/L acetic acid (Fujifilm Wako Chemicals, Osaka, Japan) (JIS special grade) was added. After 30 min, the mixture was centrifuged, the supernatant was removed, 1 mL of DDH_2_O was added, and the mixture was washed twice. Following that, the samples were dried using a dry block incubator (Bio Medical Sciences, Tokyo, Japan) at 52 °C for 96 h. All the centrifugation steps were conducted at 10 000 rpm for 1 min [6, 21, 23].

### Isotope analysis

For isotope analysis, a Gas Bench II (Thermo Fisher Scientific, Waltham, MA, USA) and a stable isotope ratio mass analyser Delta V plus (Thermo Fisher Scientific) were used. Prior to analysis, 1.4 mg of each sample and 0.07 mg of each standard [NBS18 (*δ*^18^O = −23.2; *δ*^13^C = −5.014) and IAEA-603 (*δ*^18^O = −2.37; *δ*^13^C = 2.46); Shoko Science, Yokohama, Japan] were weighed into individual 4.5 mL vials (Labco Ltd, London, UK). The samples were then purged with He gas (flow rate: 85–100 mL/min), dehydrated, and reacted for 24 h at 25 °C on a temperature-controlled tray after adding 5–6 drops of dehydrated phosphoric acid (density: >1.92 g/cm^3^). The isotope ratio is expressed as per mil (‰) using the *δ* notation, with Vienna Pee Dee Belemnite (VPDB) carbonate as the reference, representing the deviation from the established standard isotope ratio for each element. This is expressed as follows:


$$ {\delta}^{18}{\mathrm{O}}_{\mathrm{samples}}=\left(\frac{\left({}^{18}\mathrm{O}/{}^{16}\mathrm{O}\right) {\mathrm{samples}}}{\left({}^{18}\mathrm{O}/{}^{16}\mathrm{O}\right) {\mathrm{standard}}}-1\right)\times 1\ 000 $$

$$ {\delta}^{13}{\mathrm{C}}_{\mathrm{samples}}=\left(\frac{\left({}^{13}\mathrm{C}/{}^{12}\mathrm{C}\right) {\mathrm{samples}}}{\left({}^{13}\mathrm{C}/{}^{12}\mathrm{C}\right) {\mathrm{standard}}}-1\right)\times 1\ 000 $$

For the measurements, the isotope ratios of standard oxygen and carbon were determined, and the isotope ratios of the samples were corrected using a two-point calibration.

#### Measurement accuracy of the samples

To investigate accuracy of multiple measurements for the same sample, the same sample was analysed three times under identical conditions. The oxygen and carbon isotope ratios in the 65 samples were compared.

#### Changes in isotope ratios due to wetting and drying during long-term storage of samples

To investigate changes in the isotope ratios due to wetting and drying during the long-term storage of samples, enamel powder obtained from the same sample was divided into two groups: the dry (+) group, where the powder was dried for 3 h in a dry block incubator before analysis, and the dry (−) group, where the preprocessed powder was stored at room temperature for 1 month before analysis without redrying. The isotope ratios of oxygen and carbon for each group were compared. Ten samples from each group were analysed.

#### Oxygen and carbon isotope ratios in 10 areas

We investigated the correlation between oxygen and carbon isotope ratios and the place of residence during enamel formation (categorized into 10 areas). Furthermore, we calculated the correlation between the oxygen isotope ratio in each sample and the latitude, temperature, and altitude effects. The annual average temperature data for 2022 were obtained from the Japan Meteorological Agency database, and the latitude and elevation data were sourced from the Geographical Survey Institute database. A total of 65 samples were analysed in this study.

### Statistical analysis

For the statistical analysis, it was inferred from previous studies that the distribution follows a normal distribution. Therefore, Student’s *t*-test, Pearson’s product–moment correlation coefficient, and Tukey’s multiple comparison test were used [20]. Statistical significance was set at *α* ≤ 0.05. The results are expressed as mean ± standard deviation (SD).

## Results

### Measurement accuracy of the samples

The results of three repeated analyses conducted using the same sample revealed no significant differences in the isotope ratios of either element. The results obtained in the three experiments are shown in Table 2.

**Table 2:** Result of investigation of measurement accuracy of the samples (mean ± SD, ‰).

**Experiment**	***δ*** ^**18**^ **O**	***δ*** ^**13**^ **C**
First	−6.34 ± 1.07	−11.67 ± 0.83
Second	−6.32 ± 1.06	−11.66 ± 0.83
Third	−6.29 ± 1.04	−11.64 ± 0.82

SD: standard deviation.

### Changes in isotope ratios due to wetting and drying during long-term sample storage

No significant differences were observed in the isotope ratios of carbon and oxygen between the dry (−) (*δ*^18^O: average, −6.92 ± 0.81‰; *δ*^13^C: average, −12.18 ± 0.43‰) and dry (+) (*δ*^18^O: average, −7.11 ± 0.79‰; *δ*^13^C: average, −12.18 ± 0.42‰) samples (Table 3).

**Table 3:** Result of investigation of changes in isotope ratios due to wetting and drying during long-term sample storage (‰).

**Sample No.**	***δ*** ^**18**^ **O**		***δ*** ^**13**^ **C**	
	**Dry (−)**	**Dry (+)**	**Dry (−)**	**Dry (+)**
1	−6.58	−6.71	−12.08	−12.11
2	−7.19	−7.38	−12.39	−12.35
3	−7.47	−7.88	−11.56	−11.62
4	−7.27	−7.58	−12.33	−12.34
5	−8.57	−8.65	−13.18	−13.15
6	−6.31	−6.48	−11.84	−11.82
7	−5.61	−5.97	−12.19	−12.23
8	−7.02	−7.26	−12.19	−12.20
9	−6.86	−6.54	−12.15	−12.16
10	−6.27	−6.67	−11.87	−11.82
Mean ± SD	−6.92 ± 0.81	−7.11 ± 0.79	−12.18 ± 0.43	−12.18 ± 0.42
*P*-value	0.59		0.99	

SD: standard deviation.

### Oxygen and carbon isotope ratios in 10 areas

The details of the analysis results are shown in Tables 4 and 5. The average oxygen isotope ratio was highest in the Okinawa area and lowest in the Hokkaido area, while the average carbon isotope ratio was highest in the Kinki area and lowest in the Hokuriku area. The oxygen isotope ratios tended to decrease as the proximity of residential areas to cold areas increased, whereas no significant differences were observed in the carbon isotope ratios among the areas.

**Table 4:** Result of investigation of oxygen and carbon isotope ratios in 10 areas (‰).

**Area**	***δ*** ^**18**^ **O**	***δ*** ^**13**^ **C**
Hokkaido	−7.92 ± 0.50	−12.45 ± 0.57
Tohoku	−6.81 ± 0.31	−11.31 ± 0.87
Kanto	−6.86 ± 0.68	−11.94 ± 0.44
Hokuriku	−7.74 ± 0.80	−12.58 ± 0.59
Tokai	−6.59 ± 0.26	−11.64 ± 1.01
Kinki	−6.26 ± 0.49	−11.07 ± 0.95
Chugoku	−6.01 ± 0.27	−11.87 ± 0.89
Shikoku	−6.22 ± 0.52	−11.63 ± 0.66
Kyusyu	−6.40 ± 0.54	−11.58 ± 0.69
Okinawa	−4.61 ± 0.58	−11.89 ± 0.73

SD: standard deviation.

**Table 5:** The results of the test using Tukey’s test determined whether there were significant differences in oxygen isotope ratios between different locations.

	**Hokkaido**	**Tohoku**	**Kanto**	**Hokuriku**	**Tokai**	**Kinki**	**Chugoku**	**Shikoku**	**Kyusyu**	**Okinawa**
Hokkaido		n.s.	0.02	n.s	0.00	0.00	0.00	0.00	0.00	0.00
Tohoku			n.s.	n.s.	n.s.	n.s.	n.s.	n.s.	n.s.	0.00
Kanto				n.s.	n.s.	n.s.	n.s.	n.s.	n.s.	0.00
Hokuriku					0.01	0.00	0.00	0.00	0.00	0.00
Tokai						n.s.	n.s.	n.s.	n.s.	0.00
Kinki							n.s.	n.s.	n.s.	0.00
Chugoku								n.s.	n.s.	0.00
Shikoku									n.s.	0.00
Kyusyu										0.00
Okinawa										

n.s.: not significant.

**Figure 1:**
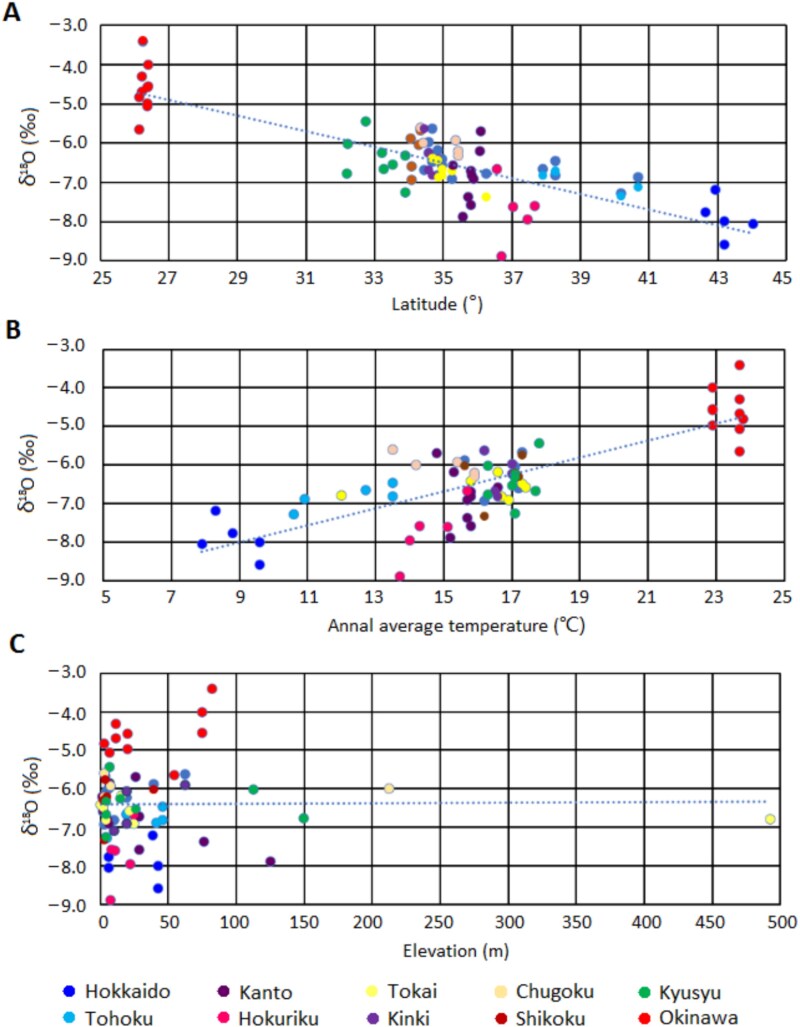
The relationships between oxygen isotope ratios in 10 areas and the (A) latitude, (B) temperature, and (C) altitude effects in each area. The oxygen isotope ratios correlated with the latitudes (*r* = −0.84), average annual temperatures (*r* = 0.81), and elevations (*r* = 0) of the residential areas of the sample providers.

Furthermore, the correlation coefficients between the oxygen isotope ratios and the latitudes, average annual temperatures, and elevations of the sample providers’ residential areas were −0.84, 0.81, and 0, respectively. Overall, the oxygen isotope ratios were correlated with the latitudes and average temperatures; however, no correlation was observed between the oxygen isotope ratios and the altitudes of the residential areas (Figure 1).

## Discussion

The oxygen isotope ratios for different areas were classified among 10 areas compared to the Hokkaido area, with significant differences observed in seven areas (Tohoku, Kanto, Tokai, Kinki, Chugoku, Shikoku, and Kyushu areas). Similarly, when compared to the Hokuriku area among the 10 classified areas, significant differences were found in 5 areas (Tokai, Kinki, Chugoku, Shikoku, and Kyushu areas). The Okinawa area, with its geographical characteristics of being at a lower latitude and having higher annual temperatures than other areas, had significantly different oxygen isotope ratios than all other areas (Tables 4 and 5). This can be attributed to the strong influences of latitudes and temperatures making the Okinawa area distinct from other areas. The oxygen isotope ratios in the Hokuriku area were lower than those in the Tohoku area which is located at a higher latitude. This is believed to be influenced by the characteristics observed in studies in which the isotopic ratios in precipitated water and groundwater were analysed in Japan. These studies indicated that the delayed replenishment of meltwater, particularly on the Sea of Japan side, leads to lower isotope ratios, whereas the Pacific Ocean side tends to exhibit higher isotope ratios. The fact that the residential areas of the Tohoku area used in this study are concentrated on the Pacific Ocean side is considered a possible reason for the low isotope ratios observed in the Hokuriku area.

Moreover, the change in the oxygen isotope ratio due to environmental changes in residential areas is estimated to be ~0‰–1‰ over a span of 6 million years, which is considered to be small within the timeframe of human existence [24]. On the other hand, although we did not observe any variations among the areas based on carbon isotope ratios, through the analysis of carbon and nitrogen isotope ratios in Jomon skeletal remains from a period around 10 000 years ago, regional differences in diet were observed. However, this study suggests that due to globalization and the development of distribution networks in modern Japan, regional differences in diet within the country have diminished. However, it has been reported that the dietary habits of Japanese and Americans during the war were very different, and clear differences in carbon isotope ratios were observed. Therefore, it is considered possible to use carbon isotope ratios to estimate the place of birth between regions with widely different diets [8, 25, 26].

In this study, the oxygen isotope ratios in the enamel bioapatite showed the strongest correlation with the latitudes of the residential areas of the sample providers (*r* = −0.84). A high correlation was found between the latitude of the sample collection location and the air temperature, suggesting that differences by latitude may depend to a small extent on temperature differences (*r* = 0.97). Furthermore, no significant correlation with the altitude was observed, as the residential areas of the sample providers were concentrated in low-elevation urban areas; however, in previous studies conducted in Europe, a slight correlation between the latitude, elevation, and oxygen isotope ratio was observed [20]. These trends are consistent within the limited range of Japan that was analysed in this study.

Stable isotope analyses using bioapatite from bones and teeth have been conducted at various research institutions. However, sample preparation methods, reaction times, and temperature effects on phosphoric acid can vary among institutions, and optimization of techniques has not been performed [27–29]. The analysis conducted using GasBench II revealed that the carbonate content in the bioapatite of human enamel varied from 2.5% to 5%. Additionally, the carbonate content showed heterogeneity among sites and individuals. Therefore, consultation with the laboratory is recommended to determine the optimal sample amount [23, 28].

In this study, we prepared the sample by grinding over the entire circumference except for the occlusal surface, and the amount of sample was set to 20 times the standard for analysis. As a result, no significant differences were observed in the repeated analyses of the samples (average standard deviations: *δ*^18^O: 0.22 ± 0.15‰, *δ*^13^C: 0.14 ± 0.10‰). Furthermore, when decayed sites were used for the stable isotope analysis, the strontium and carbon isotope ratios were not significantly affected. However, previous reports have suggested that the oxygen isotope ratio in decayed sites increases compared with that in healthy dental enamel. Therefore, decayed teeth were excluded from the study [16].

Although the oxygen isotope content of samples changes due to wetting and drying during sample storage [21], our results suggest that changes do not occur after 1 month of storage in terms of wetting and drying. These results suggested that leaving the samples for 1 month after pretreatment would not affect the results of the analysis. However, it is not clear exactly how long this would affect the analysis results, so further studies are needed. In addition, differences were observed between Okinawa areas and the other areas based on oxygen isotope ratios. However, there were also areas where no differences were observed between areas. Even in comparisons with areas outside Japan where environmental factors such as latitude, temperature, and altitude are similar, there is a possibility that significant differences may not be observed based solely on oxygen isotope ratios [16, 17].

Furthermore, oxygen isotopes in bioapatite are thought to be significantly influenced by what the body takes in as drinking water. However, in modern society, the prevalence of bottled mineral water consumption has increased, which is expected to lead to a homogenization of oxygen isotope ratios in apatite. However, it is not yet clear to what extent the oxygen taken in through food moisture and respiration influences the oxygen isotope ratios in bioapatite. Further investigation is necessary in this regard.

This study is also expected to be applied to the identification of remains between Japan and regions with different latitudes and temperatures. In the southern regions, in particular, the local population shares morphological characteristics similar to those of the Japanese population, making identification based on these features challenging. Additionally, DNA analysis can be difficult owing to degradation attributed to heat, ultraviolet radiation, and acidic soils. In such cases, stable isotope analysis can be conducted to determine the place of residence during enamel formation. This study suggests that analysing the oxygen isotope ratios in enamel bioapatite is useful for estimating the place of residence during enamel formation in Japan.

In future studies, it will be necessary to increase the number of samples in areas where no significant differences were observed in this study. Additionally, combining the analysis of isotopes of other elements and comparing the oxygen isotope ratios of the local people in southern areas with those of Japanese individuals will be essential. This comparative analysis, along with the findings of this study, will provide a more comprehensive understanding.
